# Elucidating the Mechanism of Action of the Attributed Immunomodulatory Role of Eltrombopag in Primary Immune Thrombocytopenia: An In Silico Approach

**DOI:** 10.3390/ijms22136907

**Published:** 2021-06-27

**Authors:** Maria L. Lozano, Cristina Segú-Vergés, Mireia Coma, María T. Álvarez-Roman, José R. González-Porras, Laura Gutiérrez, David Valcárcel, Nora Butta

**Affiliations:** 1 Hospital General Universitario Morales Meseguer, Centro Regional de Hemodonación, Universidad de Murcia, IMIB-Arrixaca, CB15/00055-CIBERER, 30007 Murcia, Spain; 2 Anaxomics Biotech S.L., Diputació 237, 1°, 1^a^, 08007 Barcelona, Spain; cristina.segu@anaxomics.com (C.S.-V.); mcoma@anaxomics.com (M.C.); 3 Unidad de Trombosis y Hemostasia, Servicio de Hematología, Hospital Universitario La Paz, Instituto de Investigación Hospital Universitario La Paz (IdiPAZ), Paseo de la Castellana 261, 28046 Madrid, Spain; talvarezroman@gmail.com; 4 Unidad de Hemostasia y Trombosis, Servicio de Hematología, Hospital Universitario de Salamanca, Instituto de Investigación Biomédica de Salamanca (IBSAL), Paseo de San Vicente, 58-182, 37007 Salamanca, Spain; jrgp@usal.es; 5 Grupo de Investigación en Plaquetas, Instituto de Investigación Sanitaria del Principado de Asturias (ISPA), Departamento de Medicina, Universidad de Oviedo, 33071 Oviedo, Spain; gutierrezglaura@uniovi.es; 6 Servicio Hematología, Vall d´Hebron Insitute of Oncology (VHIO), Hospital Univesitario Vall d’Hebron, Universitat Autònoma de Barcelona, Centro Cellex, Natzaret, 115-117, 08035 Barcelona, Spain; dvalcarcel@vhio.net; 7 Instituto de Investigación HospitaUniversitario La Paz (IdiPAZ), Paseo de la Castellana 261, 28046 Madrid, Spain

**Keywords:** eltrombopag, primary immune thrombocytopenia, immunomodulation, in silico, systems biology, mathematical modelling, artificial intelligence

## Abstract

Eltrombopag is a thrombopoietin receptor (MPL) agonist approved for the treatment of primary immune thrombocytopenia (ITP). Recent evidence shows that some patients may sustain platelet counts following eltrombopag discontinuation. The systemic immunomodulatory response that resolves ITP in some patients could result from an increase in platelet mass, caused either by the direct action of eltrombopag on megakaryocytes through MPL stimulation, or potential MPL-independent actions on other cell types. To uncover the possible mechanisms of action of eltrombopag, in silico analyses were performed, including a systems biology-based approach, a therapeutic performance mapping system, and structural analyses. Through manual curation of the available bibliography, 56 key proteins were identified and integrated into the ITP interactome analysis. Mathematical models (94.92% mean accuracy) were obtained to elucidate potential MPL-dependent pathways in non-megakaryocytic cell subtypes. In addition to the effects on megakaryocytes and platelet numbers, the results were consistent with MPL-mediated effects on other cells, which could involve interferon-gamma, transforming growth factor-beta, peroxisome proliferator-activated receptor-gamma, and forkhead box protein P3 pathways. Structural analyses indicated that effects on three apoptosis-related proteins (BCL2L1, BCL2, BAX) from the Bcl-2 family may be off-target effects of eltrombopag. In conclusion, this study proposes new hypotheses regarding the immunomodulatory functions of eltrombopag in patients with ITP.

## 1. Introduction

Primary immune thrombocytopenia (ITP) is an autoimmune disease defined by a low platelet count (<100 × 10^9^/L) caused by platelet destruction and impaired platelet production [1,2]. In addition, in most patients, the levels of the hormone thrombopoietin (TPO)—the major physiological regulator of megakaryocyte proliferation and platelet production— are not elevated, as could be expected due to the reduction in platelet-megakaryocyte mass [3,4]. Clinical manifestations of ITP patients are diverse and therapeutical treatments are reserved for those with bleeding or a bleeding risk [5]. It is highly recommended that in adults who are corticosteroid-dependent or have no response to corticosteroids, medical therapies be used that have robust clinical trial evidence, such as the thrombopoietin receptor agonists (TPO-RAs) rituximab or fostamatinib [6]. Eltrombopag is one of the TPO-RAs approved for the treatment of ITP that binds to the transmembrane domain of the thrombopoietin receptor (MPL) and initiates the signaling cascades (JAK/STAT and MAPK) that are similar but not identical to those of endogenous TPO, that promote proliferation and differentiation from bone marrow progenitor cells to increase platelet production [7,8]. Moreover, MPL is also expressed on hemangioblasts and hematopoietic stem cells [9]. In that sense, this drug is also approved for the treatment of severe aplastic anemia [8].

On the basis of the mechanism of action (MoA) of the drug, patients were thought to require continuous eltrombopag therapy to maintain adequate platelet counts; however, during the last decade, there have been several reports of sustained platelet responses in a percentage of ITP patients after eltrombopag or romiplostim (another TPO-RA) discontinuation (Table S1). Two small prospective studies identified around 30% of patients who were able to completely discontinue eltrombopag for at least 12 weeks while maintaining hemostatic platelet counts [10,11]. These rates of therapy-free responses (TFRs) are in line with those reported in adult and pediatric patients treated with TPO-RA [12,13,14,15,16,17,18,19,20,21,22]. Although to date we still cannot identify on which patients this approach is likely to be most successful, a complete response to low doses of TPO-RA and a diagnosis of ITP < 1 year could lead to higher TFRs [15,23]. Currently, there are three prospective clinical trials aiming to evaluate TFRs in adults with ITP that have completed the inclusion of patients [24], and final findings have not yet been reported for two of them: (i) *Rate of Prolonged Response After Stopping TPO-RA Treatment in ITP (STOP-AGO)* (ClinicalTrials.gov Identifier: NCT03119974), and (ii) *A Study to Assess the Ability of Eltrombopag to Induce Sustained Remission in Subjects with ITP (TAPER)* (ClinicalTrials.gov Identifier: NCT03524612).

In the face of these compelling data, deciphering the actual mechanism through which eltrombopag may elicit these responses, which may be MPL- and/or off-target mediated, would certainly be of great interest. To date, the evidence points towards two hypotheses. Currently, the most widely accepted presumption implies that the increase in platelet counts mediated by eltrombopag would play an immunomodulatory role, associated with an increase in regulatory CD4+ T cells and a reduction in effector T-cell helper functions. Moreover, TPO-RA could restore immune tolerance to platelets by increasing the exposure to platelet antigens [3,25,26,27], an effect that eltrombopag, contrary to romiplostim, exerts through its binding to the transmembrane domain of the receptor. Furthermore, eltrombopag has been reported to bind exclusively to human and primate MPL [28]; however, it has been found to exert a response in murine models [29,30], which indicates the existence of MPL-independent mechanisms for eltrombopag. Thus, alternatively, off-target effects of eltrombopag may also be involved, including MPL-mediated and MPL-independent mechanisms affecting other immune cells [31] and MPL-independent stimulation of hematopoietic stem cells. In this sense, it has been shown that eltrombopag can chelate iron and that this chelating action enables it to improve the function of bone marrow stem cells [32].

Upon TPO binding to this receptor, a wide array of downstream signaling pathways are activated, promoting cellular survival and proliferation. Functional alterations of MPL are associated with defects in platelet counts and with aplastic anemia. MPL, which was discovered as the human homologue of an oncogene identified from the murine myeloproliferative leukemia virus, has been portrayed as a critical oncogenic protein in recent studies [33,34,35,36]. Additionally, the RNA sequencing analysis of different blood subsets has revealed that MPL is not only expressed in the megakaryocytic/platelet fraction but is also present in some immune cell subpopulations, with CD4-naïve regulatory T cells (Tregs) being the ones that express it most abundantly [37]. MPL gene expression has been identified in other cell subsets, such as B lymphocytes [38], CD4 and CD8 T cells [37,38], monocytes [37,38], neutrophils [38], and NK cells [37].

The evaluation of the initiation and maintenance of immunotolerance following eltrombopag therapy is challenging, mainly because of the complex regulatory processes and the diversity and overlapping immune components that may collectively be involved in these responses. Systems biology methods are an increasingly recurring strategy to understand the molecular effects of a drug in complex clinical settings [39,40,41]; to identify diagnoses, prognoses, or response markers [42,43]; or to develop strategies for novel treatments [44,45]. Altogether, systems biology and in silico techniques are another element to be added to the toolbox when trying to unveil the mechanisms that lie behind complex clinical observations.

In this study, we used an in silico approach based on systems biology and structural analyses to explore the potential immunomodulatory MoA of eltrombopag in the treatment of ITP. We created mathematical models of the underlying protein pathways to explain biological outcomes beyond the ones stemming merely from the increase in platelet counts. Because these are theoretical models, it is important to note that they are not necessarily associated with clinical effects in real patients, but they still point towards potential mechanisms that could explain these effects.

## 2. Results

### 2.1. Primary Immune Thrombocytopenia Interactome

**Figure 1:**
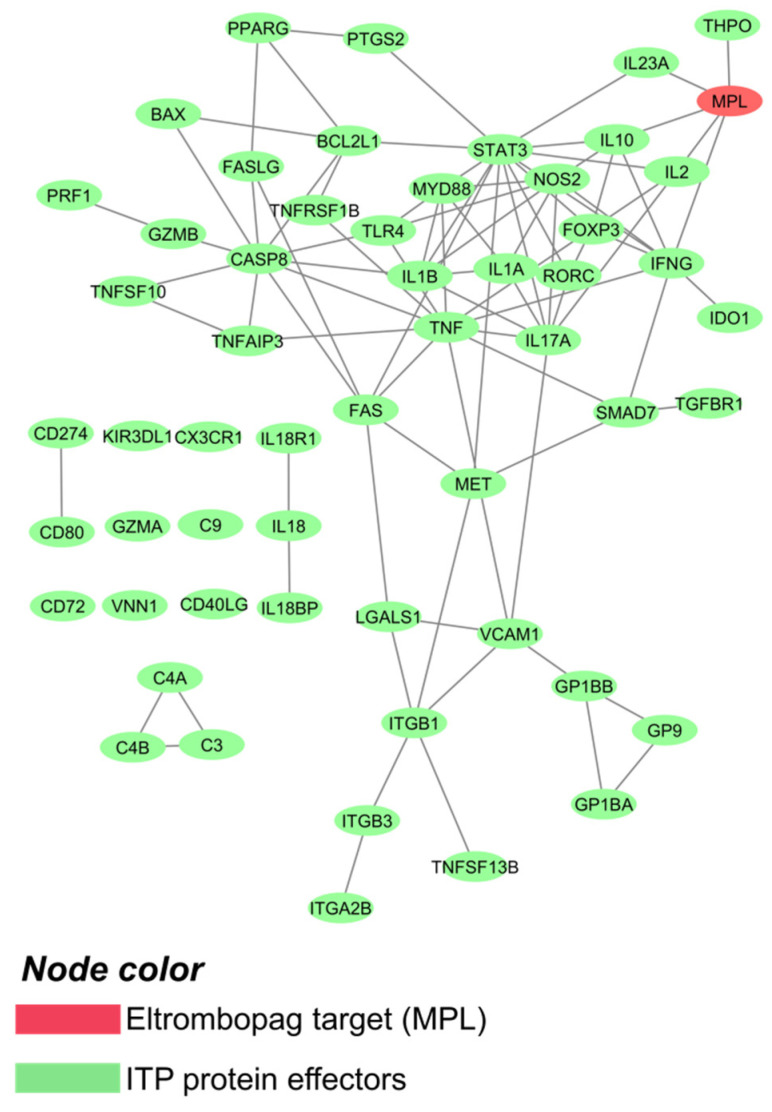
ITP interactome, focusing on drug targets and disease main players. Image created with Cytoscape3.6 [46].

Through the curation of the currently available bibliography on ITP, five ITP pathophysiological mechanisms (motives) were identified: (i) an abnormal B-cell-dependent humoral immune response, (ii) abnormal cellular immunity, (iii) immune-induced platelet destruction, (iv) suppression of megakaryocyte proliferation and maturation/decreased megakaryocyte apoptosis, and (v) dysfunctional mesenchymal stem cells (Table S2). Subsequently, each motive was functionally characterized at the protein level, based on corresponding molecular effectors. A total of 56 key proteins were identified and used as the base for the analysis within the human protein network (Table S3), namely, the ITP interactome. The interactome around ITP key proteins comprised 2756 proteins (Table S4) and 49,081 interactions (Figure S1). MPL, eltrombopag’s protein target, was found to be directly connected to ITP key proteins (Figure 1).

### 2.2. Eltrombopag Could Increase TGF-β Expression through MPL Signaling

**Figure 2:**
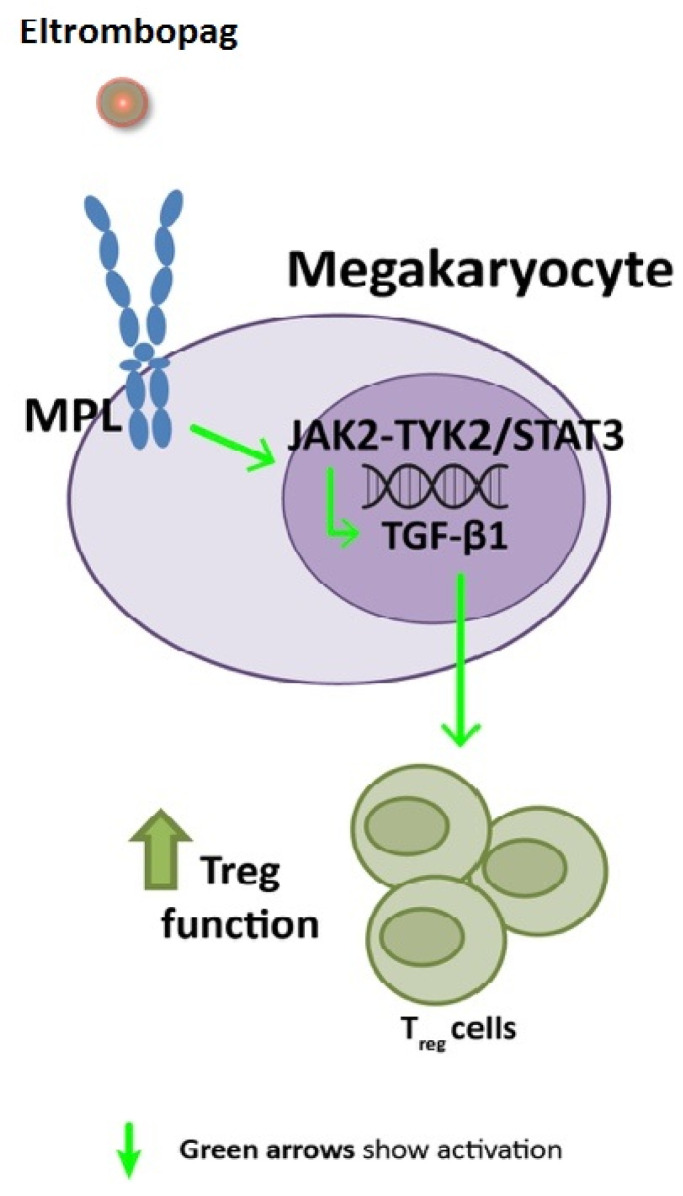
Predicted mechanism of action of eltrombopag affecting TGF-β expression in primary immune thrombocytopenia megakaryocytes (or other cell types). Model path outputs can be found in Supplementary Figure S2A and scientific literature supporting the predicted mechanisms can be found in Supplementary Table S5. MPL: thrombopoietin receptor; TGF-β: transforming growth factor-beta. MPL: thrombopoietin receptor; TGF-β: transforming growth factor-beta.

Sampling-based methods were generated to build a set of ITP mathematical model solutions with a mean cross-validated accuracy of 94.92%; these models were used to elucidate the MoA of eltrombopag in ITP, and specifically its immunomodulatory effects. The evaluation of ITP sampling-based mathematical models allowed the identification of the most probable, biologically plausible protein paths connecting MPL to ITP molecular key proteins. These results show that the interaction between eltrombopag and MPL could activate the Janus kinase family, mainly the tyrosine-protein kinase JAK2 and the non-receptor tyrosine-protein kinase TYK2. Both of these could induce the activation of STAT3, which may upregulate the expression of transforming growth factor-beta (TGF-β). Since many types of cells synthesize TGF-β, this increased production of TGF-β mediated by MPL engagement might not be megakaryocyte-specific; if other immune cells express the receptor and its downstream signaling molecules, this pathway could also be relevant in increasing the levels of this cytokine (Figure 2).

### 2.3. Signaling of Eltrombopag through MPL on Immune Cells Could Affect IFN-γ, PPARγ, and FOXP3 Function

**Figure 3:**
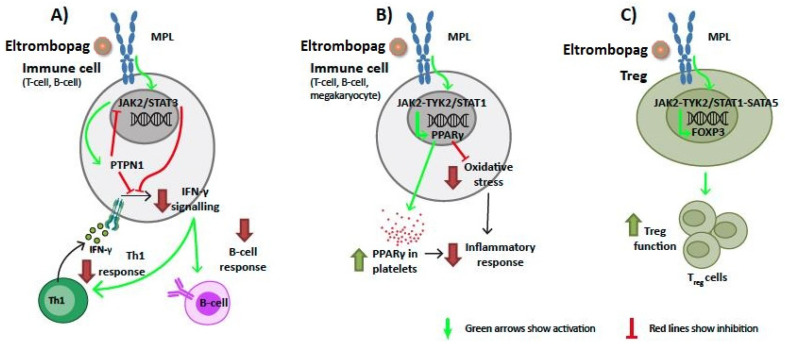
Predicted mechanism of action of eltrombopag affecting (**A**) IFN-γ signaling; (**B**) PPARγ expression; and (**C**) FOXP3 activity in primary immune thrombocytopenia immune cells. Model path outputs can be found in Supplementary Figure S2B–D and scientific literature supporting the predicted mechanisms can be found in Supplementary Table S5. FOXP3: forkhead box protein P3; IFN-γ: interferon-gamma; MPL: thrombopoietin receptor; PPARγ: peroxisome proliferator-activated receptor-gamma; PTPN1: tyrosine-protein phosphatase non-receptor type 1.

According to ITP mathematical models, MPL signaling through the activated JAK2-STAT3 pathway could induce inhibition of the signaling of the pro-inflammatory cytokine interferon-gamma (IFN-γ) in B cells and T cells, either directly or through the activation of tyrosine-protein phosphatase non-receptor type 1 (PTPN1) (Figure 3A). However, at the same time, PTPN1 can act as a regulator of this pathway through negative feedback, inhibiting JAK-STAT signaling. Moreover, the combined activity of JAK2 and TYK2 on STAT1 could stimulate the expression of peroxisome proliferator-activated receptor-gamma (PPARγ), which has been associated with a reduction of oxidative stress and inflammation processes (Figure 3B). Additionally, both JAK2 and TYK2 could upregulate, through STAT1 and STAT5, the expression of forkhead box protein P3 (FOXP3), involved in the differentiation of Tregs (Figure 3C).

### 2.4. Eltrombopag Could Interact with the BCL2 Protein Family

We applied the chemocentric approach, which assumes that two similar molecules probably have similar properties (either sharing the same biological targets or showing a similar pharmacological profile), to identify in silico chemicals with eltrombopag-like structures (Tanimoto index > 80%). We retrieved one similar structure, CHEMBL3417402, which the three compound-target databases (PubChem [47,48], ChEMBL [49], and Binding [50]) reported to have active bioactivity profiles and to act on the same known human targets. Thus, we identified three target proteins as potential ligands for eltrombopag, all of which were associated with apoptosis modulation and related to B-cell lymphoma 2 (Bcl-2), namely: BCL-2-like protein 1 (BCL2L1), apoptosis regulator BCL-2 (BCL2), and apoptosis regulator BAX (BAX).

We then performed docking studies, using a computation method to confirm, through structural analysis, the ability of eltrombopag to bind and interact with the off-target candidates. Relevant functional sites of the protein were explored to evaluate the affinity of the interactions between the drug and the candidate-protein (Table 1).

**Figure 4:**
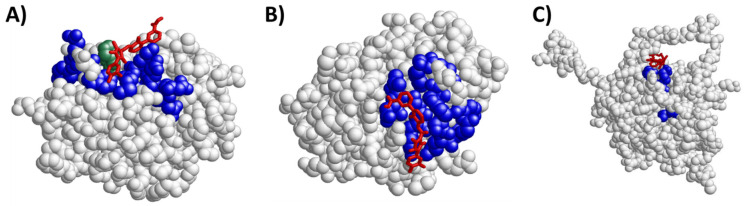
Structures from docking results for the predicted new targets (representing atoms as balls) and eltrombopag (structure represented as red sticks). (**A**) Bcl-2: amino acids highlighted in blue correspond to the BH4 domain and in green to histidine 20—Protein Data Bank identification code: 2XA0 [51]. (**B**) BCL2L1: amino acids highlighted in blue correspond to the BH4 domain—Protein Data Bank identification code: 1MAZ [53]. (**C**) BAX: amino acids highlighted in blue correspond to amino acids 60, 61, 64, and 70 from the BH3 domain—Protein Data Bank identification code: 2K7W [55]. Images were created with the software RasMol [57]. Bcl-2: B-cell lymphoma 2; BCL2L1: Bcl-2-like protein 1; BH3: Bcl-2 homology 3; BH4: Bcl-2 homology 4.

The three potential targets tested were all validated and potential pockets were found within the thresholds of energy and distance that had been previously established. The predicted major binding sites within BCL2 and BCL2L1 for eltrombopag included the whole Bcl-2 homology (BH)4 domain, some particular amino acids in BH4, and the histidines in BH4. In the case of BAX, the predicted docking sites comprised the alpha helix-1, the whole BH3 domain, and some particular amino acids in either alpha helix-1 or the BH3 domain (Figure 4).

## 3. Discussion

**Figure 5:**
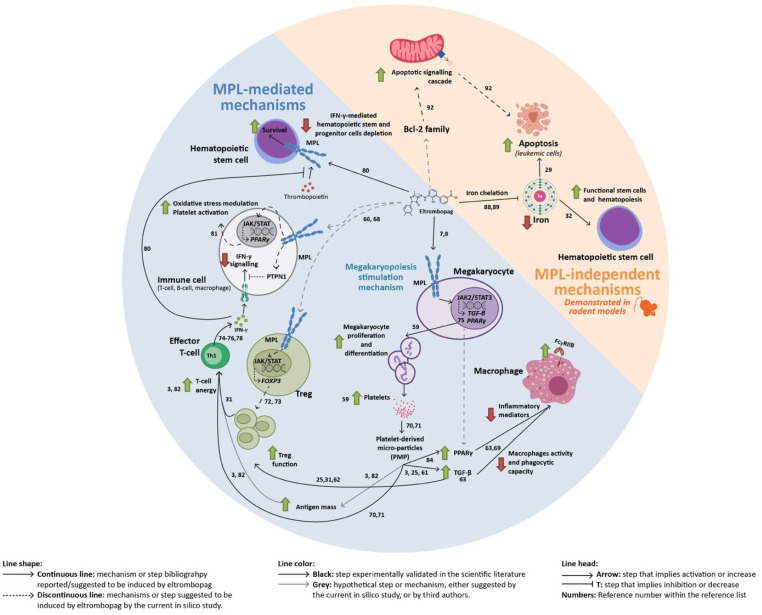
Summary of the immunomodulatory mechanisms described and hypothesized for eltrombopag. Bcl-2: B-cell lymphoma 2; FOXP3: forkhead box protein P3; IFN-γ: interferon-gamma; MPL: thrombopoietin receptor; PMP: platelet-derived microparticles; PPARγ: peroxisome proliferator-activated receptor gamma; PTPN1: tyrosine-protein phosphatase non-receptor type 1; TGF-β: transforming growth factor beta; Treg: regulatory T cell.

The growing evidence highlighting that eltrombopag can trigger TFR in ITP patients suggests an immunomodulatory activity of the drug (Table S1). However, the hypotheses put forward to explain this effect have not reached a global consensus, propelling investigations to unravel the mechanisms through which eltrombopag acts in these particular cases. Herein, we have applied in silico systems biology and structural-based approaches with the aim of exploring eltrombopag’s off-target effects (understood as MPL-mediated effects on non-megakaryocyte cells or mechanisms that are MPL-independent) that could explain novel ITP immune-related pathophysiological pathways. According to our in silico analysis, data indicate that eltrombopag’s MoA could be mediated by either direct signaling on MPL (affecting megakaryocytes and potentially other cells) or by indirect (non–MPL-mediated) effects. These results have been contextualized within the suggested immunomodulatory mechanisms of the drug (Figure 5).

Both eltrombopag and romiplostim are known to directly stimulate megakaryopoiesis and platelet generation through pathways that are different from each other [58]. In general, the most widely accepted hypothesis of TPO-RA immunomodulation is a restoration of antigen-specific-tolerance through a direct increase in platelet counts [31,58,59]. Additionally, a regulatory loop in megakaryocytes in the bone marrow between TPO and the well-known anti-inflammatory cytokine TGF-β has been previously described [60]. Furthermore, given that platelets are the biggest reservoirs of TGF-β in the body, the increase in platelet counts would also entail an increase in this anti-inflammatory cytokine [3,61], which may empower Tregs with the responsibility and burden of maintaining homeostasis and promoting immune tolerance [25,62]. Our results suggest that stimulating MPL may not only increase the release of the TGF-β reservoir by means of the boost in platelet mass, but may also stimulate cells to produce more TGF-β, as experimentally shown in bone marrow megakaryocytes [60]. Previous data in patients treated with eltrombopag showed an increase in TGF-β that correlated with the drug response [63,64]. TGF-β was shown to positively correlate with soluble cytotoxic T-lymphocyte-associated antigen 4 (sCTLA-4), which by itself can modulate and terminate the immune response [64]. This cytokine, synthesized by most hematopoietic cell subtypes, including Tregs, is a key player in the downregulation of autoreactive T cells [3] and is also important in the differentiation of Tregs and pro-inflammatory Th17 cells from CD4+ T cells [31]. Stimuli, such as phosphatidylserine exposure on apoptotic cells, have been shown to induce the production of TGF-β and promote anti-inflammatory responses [65]; our study suggests that the engagement of MPL by eltrombopag may directly contribute to the generation of this important biological mediator. This induction of TGF-β expression could not stem from hematopoietic progenitors or megakaryocytes alone, since MPL mRNA has been shown to be present in different tissues [66,67,68] and in peripheral blood populations of myeloid and lymphoid origin (including Tregs and B cells) [37,38].

In addition to Treg regulation, there is also evidence of the effect of TPO-RA on the phagocytic capacity of monocyte-derived macrophages, more specifically on the FcγR balance (in favor of the inhibitory FcγRIIb, characteristic of pro-tolerogenic phenotypes), which has also been suggested to be related to increased TGF-β levels [69].

Another hypothesis concerning the modulatory action of eltrombopag on Treg biology involves platelet-derived microparticles (PMP) [70,71]. Ectosomes (a type of microparticle) derived from platelets could interact with CD4+ T cells to diminish the release of pro-inflammatory cytokines (IFN-γ, tumor necrosis factor-alpha (TNF-α), and interleukin-6 (IL-6)). Furthermore, platelet ectosomes expressing TGF-β could induce the de novo differentiation of naïve T cells into Tregs and the conversion of memory T cells into FOXP3+ Tregs. These induced Tregs could then produce suppressive cytokines (TGF-β and IL-10) and decrease the levels of IL-2 to dampen effector T cell responses. Therefore, platelet-derived ectosomes may contribute to Treg homeostasis and hence to the level of immune tolerance.

Our system predicted some additional pathways that could modulate FOXP3, IFN-γ, and PPARγ, directing the immune response towards the induction of tolerance and termination of the abnormal immune response. Firstly, FOXP3, an essential transcription factor for Tregs, could play a key role in the suppression of the dysregulated immune response associated with ITP by inducing the differentiation of T-cell precursors to Tregs [72,73]. According to our analysis, eltrombopag would upregulate the expression of FOXP3 by modulating MPL signaling through the JAK2 or TYK2 pathways via STAT1 and STAT5. This would ultimately lead to an increase in Tregs that would exert an immunosuppressive effect to help restore immune homeostasis. Secondly, IFN-γ, a pro-inflammatory cytokine that is key in many autoimmune diseases and is highly expressed in Th1-lymphocytes and natural killer cells, is upregulated in ITP because of the characteristic Th1/Th2 imbalance of the disease [74,75,76]. Moreover, IFN-γ has been associated with the destruction of platelets in ITP [77]. Expression data from previous studies have indicated an increase in IFN-γ [78] and the IFN-γ response in ITP patients [79]. Our ITP mathematical model suggests that eltrombopag could diminish IFN-γ signaling through PTPN1, thus reducing the enhanced IFN-γ-induced response. This blockade would then antagonize the pathological immunostimulation observed in this disease. Notably, IFN-γ has been reported to block TPO:MPL complex formation and TPO-dependent MPL activation in an IFN-γ-receptor-independent manner, and eltrombopag has been proven to bypass this IFN-γ blockade of MPL binding and activation [80], a critical step in maintaining hematopoietic stem cell survival. The mechanism herein proposed, involving PTPN1, would be mediated by the binding of eltrombopag to MPL and subsequent IFN-γ signaling disruption and would therefore be distinct from the inability of IFN-γ to disrupt the eltrombopag–MPL interaction through steric occlusion [80]. Thirdly, vanin-1, an oxidative stress sensor in epithelial and blood mononuclear cells, has been identified as a marker for chronic ITP [81,82]. In addition, microRNAs differentially expressed in T cells from ITP patients regulate the activity of the vanin-1 gene [83]. The upregulation of vanin-1 in response to oxidative stress is associated with a downregulation of PPARγ [81]. These data indicate that the oxidative stress–vanin-1–PPARγ axis contributes to ITP’s immune dysfunction and to the suboptimal response to treatment [81]. Aside from the role of PPARγ on immune cells, this protein has also been found to be expressed in platelets and megakaryocytes and has been proposed to play a relevant role in platelet activation [84]. In fact, this transcription factor has been found in platelet-derived microparticles [85]. Altered platelet-derived microparticles containing PPARγ may induce monocyte signaling pathways in a transcellular fashion [86]. Our results suggest that eltrombopag binding to MPL could lead to PPARγ upregulation in megakaryocytes or other immune cells, and could thus induce a reduction in oxidative stress and pro-inflammatory environment.

However, these approaches are limited by the information available about drugs and diseases, so in our study, some assumptions had to be made: (i) most of the results relied on the expression and activity of MPL on non-megakaryocyte cell types, and (ii) our evaluation was made with the pathophysiological mechanisms of ITP described at the moment of the study, which could be improved in the future. The fact that MPL mRNA has been found in non-megakaryocytic cells [37,38,66,67,68], although not necessarily indicating MPL protein expression [87], may support the contribution, through these signaling processes, of other cell subsets to the immunomodulatory effects of the drug. The most plausible explanation in the literature is related to an increase in the platelet mass that could indirectly affect the production of antibodies through the induction of T cell anergy and thus facilitate tolerance [3,82]. Similarly to indirect effect of eltrombopag proposed here, this theory has also not yet been proven.

Eltrombopag is species-specific and only binds to human or chimpanzee MPL [28]. Some of its effects have been previously demonstrated to be exerted by indirect, non-MPL mediated mechanisms. For example, as an iron chelator with a potential impairment on iron-induced reactive oxygen species, eltrombopag improves hematopoiesis regulation [32,88,89]. Additionally, by sequestering intracellular iron, the drug has displayed an ability to inhibit leukemia cell growth in in vitro and in vivo mouse models [29], and to stimulate hematopoiesis at the stem cell level in murine and human cells through iron chelation-mediated molecular reprogramming [32].

In the current study we have identified, through a structural computational strategy, members of the Bcl-2 family (i.e. BCL2, BAX, and BCL2L1) that are likely to be potential targets of eltrombopag. In support of the validity of this in silico analysis, it has been reported that eltrombopag directly binds and inhibits BAX, preventing cell death [90]. Platelet survival and life span in vivo are known to be regulated by members of the Bcl-2 family of pro- and anti-apoptotic proteins [91], and these proteins are also related with megakaryocyte apoptosis and function [92]. In general, TPO-RAs have been described as agents that may decrease platelet apoptosis, a mechanism that seems to have only a short-lived effect, lasting for only 14 days [93]. However, platelets from patients with ITP on long-term treatment with TPO-RA show enhanced apoptosis compared with those from untreated ITP patients [94,95,96]. This observation suggests that the effect of TPO-RA is dependent on the extent or duration of treatment. Although the effect of eltrombopag on the apoptosis of lymphocytes has not been analyzed, eltrombopag could also be involved in lymphocyte apoptosis, thus arguing for the implication of these target proteins in the immunomodulatory mechanism of the drug.

Notwithstanding these results, the protein screening protocol used for the off-target identification in the current study presents some limitations. Firstly, the list of candidates was obtained from a selection of three databases that may have not contained all available, complete, and updated data. Secondly, the docking exercise suggested that eltrombopag might interact with the proteins in functionally relevant sites, but this did not necessarily imply that the potential interaction had an effect on the function of the protein.

## 4. Materials and Methods

### 4.1. TPMS Technology: Systems Biology-Based Model Creation

The therapeutic performance mapping system (TPMS) [97] (Anaxomics Biotech, Barcelona, Spain) is a top-down systems biology approach based on artificial intelligence and pattern recognition models that integrate all available biological, pharmacological, and medical knowledge to create mathematical models that simulate the behavior of human physiology in silico. This technology relies on the protein-based human biological network and human physiology rules provided by clinical experience (extended explanation and references in Supplementary Methods).

### 4.2. Human Biological Network and Molecular Definition of Clinical Concepts

We created ITP mathematical models based on a protein–protein interaction (PPI) human network, which includes different types of relationships between proteins, including physical and functional activity and transcriptional regulation (Table S6 and Supplementary Methods). To center the analysis on the clinical concepts of interest, ITP pathophysiology and the targets of eltrombopag were molecularly defined through an in-depth analysis of bibliographical sources obtained from PubMed and manual identification of molecular effectors or key proteins (Tables S2, S3 and S7 and Supplementary Methods). The network around ITP was evaluated to ensure its connectivity to eltrombopag targets.

### 4.3. Human Physiological Rules and Mechanism Of Action Models

We used sampling-based methods [97](Supplementary Methods) to simulate the ITP models. To obtain mathematical models reflecting human physiology, the PPI network was trained using artificial intelligence approaches (Supplementary Methods) to comply with a set of known human physiological rules (Table S6), namely, relationships between drugs, defined as their protein targets, and clinical conditions—indications and adverse drug reactions—defined as proteins involved in these conditions. At this point, each link in the protein network is assigned a weight to propagate the signal from drug targets to proteins involved in diseases, forming paths of proteins and links between them with varying probabilities, in order to allow the model to behave according to human physiology. The number of drug–pathophysiology entries in the training set is smaller than the number of parameters (link weights) required by the algorithm; thus, any process modelled by TPMS considers a population of different solutions. Only solutions with accuracy > 90% (percentage of compliance of all drug–pathophysiology relationships included in the training set) were considered as valid. Thus, a universe of 100 MoA solutions that incorporated the ability of eltrombopag to interact with or influence ITP molecular players was modeled, which were analyzed as previously described [40,45,97,98,99,100]. By doing this, we aimed to obtain the more plausible paths between eltrombopag targets and ITP key proteins, both from a probabilistic and biological point of view, as per the model training. The accuracy of the final MoA model was calculated as the mean of the accuracies of all considered solutions. The MoA was biologically validated in a two-step process. Firstly, we verified that links were accurate, i.e. already described in the literature. Secondly, we evaluated whether the MoA was logical as a whole, featuring pathways coherent with the living system and the known pathophysiology of ITP.

### 4.4. Chemical Similarity

We retrieved eltrombopag’s structure-data file from PubChem (CID 9846180) [101]. We obtained protein targets associated with similar structures (Tanimoto index > 80% [102]) from publicly available databases (PubChem [47,48], ChEMBL [49], and Binding [50]) and we considered ‘candidates for interaction with eltrombopag’ to be all human proteins for which at least one similar drug presented bioactivity, as reported by the three databases.

### 4.5. Docking Analysis

We used the AutoDock Vina algorithm [103] to evaluate the potential binding of eltrombopag to its ‘candidates for interaction’ using the available crystalized structures in RCSB PDB (www.rcsb.org accessed on 28 May 2021) [104]. Biologically relevant sites were selected for each candidate structure (Supplementary Methods). The complete details of the structures and sites used can be found in Table 1. Docking results were considered positive when they presented a free energy of binding <−6 kcal/mol and a distance <5 Å.

## 5. Conclusions

There is a growing amount of data suggesting that eltrombopag may sustain the restoration of the immune cell populations of the host. Although the scenario is complex, and multiple mechanisms may be involved in the re-establishment of the immune equilibrium, this study presents some hypotheses that point towards direct effects on immune system cells and even on other proteins apart from MPL, as well as suggests some other immunomodulatory effects mediated by action on megakaryocytes, which need to be explored and validated in the future.

## Figures and Tables

**Table 1:** Structures and pockets used for docking evaluation.

Candidate Name	UniProt	PDB	Chain	Positions PDB	Pockets (Aminoacid Positions)	Type of Pocket
**BCL2**	P10415	2XA0 [51]	A	1–207	10;11;12;13;14;15;16;17;18;19;20;21;22;23;24;25;26;27;28;29;30	BH4 domain (entire), individual BH4 amino acids, histidines BH4-domain
					3;20;94;184;186	Individual histidines
			B	1–207	10;11;12;13;14;15;16;17;18;19;20;21;22;23;24;25;26;27;28;29;30	BH4 domain (entire), individual BH4 amino acids, histidines BH4-domain
					3;20;94;184;186	Individual histidines
		5FCG [52]	A	1–207	12;13;14;15;16;17;18;19;20;21;22;23;24;25;26;27;28;29;30;31;32	BH4 domain (entire), individual BH4 amino acids, histidines BH4-domain
					6;23;56;82;146;148	Individual histidines
**BCL2L1**	Q07817	1MAZ [53]	A	1–209	8;9;10;11;12;13;14;15;16;17;18;19;20;21;22;23;24;25;26;27;28	BH4 domain (entire), individual BH4 amino acids
					62;75;117;181	Individual histidines
		1R2D [54]	A	1–211	4;5;6;7;8;9;10;11;12;13;14;15;16;17;18;19;20;21;22;23;24	BH4 domain (entire), individual BH4 amino acids
					58;71;113;117	Individual histidines
**BAX**	Q07812	2K7W [55]	A	1–192	14;15;16;17;18;19;20;21;22;23;24;25;26;27;28;29;30;31;32;33;34;35;36;37;38	Alpha helix-1, individual Alpha helix-1 amino acids
					59;60;61;62;63;64;65;66;67;68;69;70;71;72;73	BH3 domain (entire), individual BH3 amino acids
		2LR1 [56]	A	1–192	14;15;16;17;18;19;20;21;22;23;24;25;26;27;28;29;30;31;32;33;34;35;36;37;38	Alpha helix-1, individual Alpha helix-1 amino acids
					59;60;61;62;63;64;65;66;67;68;69;70;71;72;73	BH3 domain (entire), individual BH3 amino acids

BAX: apoptosis regulator BAX; Bcl-2: B-cell lymphoma 2; BCL2: apoptosis regulator Bcl-2; BCL2L1: Bcl-2-like protein 1; BH4: Bcl-2 homology 4.

## Data Availability

Full data available upon reasonable request.

## Supplementary Materials

The following are available online at https://www.mdpi.com/article/10.3390/ijms22136907/s1: Supplementary Tables (Tables S1–S7); Supplementary Figures, Figure S1: ITP interactome. Protein–protein interaction network around ITP key proteins; Figure S2: Schematic representation of predicted eltrombopag mechanisms of action affecting ITP key proteins (linked to their functions in ITP); Supplementary Methods.
