# Supplementary material for: Elucidating the Mechanism of Action of the Attributed Immunomodulatory Role of Eltrombopag in Primary Immune Thrombocytopenia: An In Silico Approach
Source: Int J Mol Sci. 2021 Jun 27;22(13):6907. doi: 10.3390/ijms22136907 (PMC8269123; doi:10.3390/ijms22136907)
Supplement: Supplementary file 1 [file ijms-22-06907-s001.zip › Supplementary Methods.pdf]

SUPPLEMENTARY METHODS

Molecular and biological characterization of ITP pathophysiology and of eltrombopag effects

Through a thorough revision of bibliographical sources, we defined a set of restrictions for characterising ITP, which were used to centre the protein network and the mathematical model on ITP. We conducted a search for reviews published in the previous ten years in the PubMed database (from 2006 to the date of the search April 5th 2017) that included the following search strings: *(("Immune thrombocytopenic purpura"[Title]) OR (ITP[Title]) OR ("Primary Immune Thrombocytopenia"[Title])) AND (pathophysiology OR "molecular pathology" OR "molecular pathogenesis")*. This search resulted in 36 relevant articles that were fully reviewed. The search was expanded using article reference lists. We identified the main pathophysiological processes described to be involved in ITP: abnormal B cell-dependent humoral immune response, abnormal cellular immunity, immune induced platelet destruction, suppression of megakaryocyte proliferation and maturation/decreased megakaryocyte apoptosis, and dysfunctional mesenchymal stem cells (Table S2, Supplementary file 1). Subsequently each pathophysiological process was further functionally characterized at protein level, considering only proteins with a demonstrated functional role in disease development or manifestation; if the evidence for a protein candidate was considered weak (e.g. change in expression without clear functional involvement), specific searches were performed to include or discard the candidate. We identified 56 non-duplicated key proteins to focus the analysis on ITP in the human biological network (Table S3, Supplementary file 1), including bibliographic references linking the proteins to ITP pathophysiology).

For the drug molecular definition, we performed a revision of dedicated databases [1–3] and of scientific literature and we identified one target (MPL) (Table S7, Supplementary file 1).

Creation of human biological networks

The protein-protein interaction (PPI) human network created incorporated the available relationships (edges or links) between proteins (nodes) from a regularly updated in-house database drawn from public sources: KEGG [4,5], REACTOME [6], INTACT [7], BIOGRID [8], HPRD [9], and TRRUST [10]. We incorporated into the biological network all the information of key proteins defined during the molecular and biological characterization and stored in relevant databases (drug targets, other disease key proteins, biomarkers…).

Generation of mathematical models – Sampling-based methods

We transformed biological maps into a mathematical model capable of reproducing existing knowledge and predicting new data. As already described elsewhere[11],therapeutic performance mapping system (TPMS) technology

uses a set of artificial intelligence algorithms to simulate the human physiology over the human biological network [12–15].

To train the models, we collated into a table (training set) a selected collection of known input-output physiological signals considered the "truths" (Table S6, Supplementary file 1)[16]. We built the training set by using a compendium of databases that provides biological and pharmacological input-output relationships (such as drug-indication pairs) [17,18]. The biological or pathological conditions included in the training set were molecularly characterized through specific scientific literature search and hand-curated assignment of proteins to the conditions, constructing a clinical-molecular database that could be used as a dictionary between clinical terms and molecular processes (i.e. the biological effectors database [BED]) [19,20]. The models had to be able to reproduce every rule contained in the training set. This approach allowed the creation of models that integrated all the available biological, pharmacological, and medical knowledge and were able to suggest mechanistic hypotheses that were consistent with actual biological processes.

We used sampling–based methods to build the models to elucidate the immunomodulatory molecular mechanism of action (MoA) of eltrombopag. TPMS sampling–based methods generate models similar to a Multilayer Perceptron of an Artificial Neural Network over the human protein network (where neurons are the proteins and the edges of the network are used to transfer the information). This methodology can be used for describing with high capability all plausible relationships between an input (or stimulus) and an output (or response). Sampling–based methods use optimization algorithms [13] to solve each parameter of the equation, i.e. the weights associated to the links between the nodes in the human protein network. In this approach, the network is limited by considering only interactions that connect drug targets with protein effectors or disease key proteins in a maximum of three steps. The values of activation (+1) and inactivation (-1) of the targets of the drugs in the training set were considered as input signals. The output results are the values of activation and inactivation of the proteins defining the phenotype (as retrieved from the BED). Each node of the protein network receives as input the output of the connected nodes in the flow direction, from targets to effectors, weighted by each link weight. The sum of inputs is transformed by a hyperbolic tangent function to generate the score of the node (neuron), which becomes the "output signal" of the current node towards the nodes. The weight parameters are obtained by the Stochastic Optimization Method based on Simulated Annealing [13], which uses probabilistic measures derived from the biological evidences to adjust network interaction types and strengths. Since the number of entries in the training set is always smaller than the number of parameters (link weights) required by the algorithm, any process modelled by TPMS considers a population of different solutions. In our case, we obtained models

complying with the information in the training set with a mean accuracy of 94.92%. In order to elucidate the immunomodulatory mechanisms of eltrombopag in ITP, drug vs. disease-specific models were created by repeating the optimization process adding the new inputs (drug characterization) and output (disease characterization). Herein, our MoA represented the mean of the solutions obtained. Firstly, we checked that each link was accurate, i.e. was already described in the literature. Secondly, we evaluated whether the MoA was logical as a whole, featuring pathways coherent with the living system and the known pathophysiology of ITP.

Selection of biologically relevant sites within structures of eltrombopag-target candidates

RCSB PDB (www.rcsb.org) [21] structures for each candidate were studied to identify drug-binding sites:

- BCL2: binding of external molecules to the BH4 domain has been reported to induce a conformational change turning BCL2 from an anti-apoptotic to a death protein [22].
- BCL2L1: we did not find references of drugs interfering in its function. However, taking into account that it belongs to the same family as the abovementioned protein, BCL2, we considered the BH4 domain as a potential functional site.
- BAX: binding of antibodies to specific regions of its pro-apoptotic BH3 domain has been reported to induce BAX activation [22]. Similarly, the first alpha helix of the protein has been found necessary for activation by the BH3-only proteins Bid and PUMA [23].

Moreover, we also considered histidine residues within the whole protein sequence of target candidates as potential binding pockets of eltrombopag, because of the high importance of MPL's His^{499} (located in its transmembrane domain) in the binding of eltrombopag [24,25].

REFERENCES

[1] Wishart DS, Wu A. Using DrugBank for In Silico Drug Exploration and Discovery. *Curr Protoc Bioinforma*. 2016;54:14 4 1-14 4 31. doi:10.1002/cpbi.1

[2] Szklarczyk D, Santos A, Von Mering C, Jensen LJ, Bork P, Kuhn M. STITCH 5: Augmenting protein-chemical interaction networks with tissue and affinity data. *Nucleic Acids Res*. 2016;44(D1):D380-D384. doi:10.1093/nar/gkv1277

[3] Hecker N, Ahmed J, von Eichborn J, et al. SuperTarget goes quantitative: Update on drug-target interactions. *Nucleic Acids Res*. 2012;40(D1):1113-1117. doi:10.1093/nar/gkr912

[4] Kanehisa M, Goto S. KEGG: Kyoto Encyclopedia of Genes and Genomes. *Nucleic Acids Res*. 2000;28(1):27-30. doi:10.1093/nar/28.1.27

[5] Kanehisa M, Furumichi M, Tanabe M, Sato Y, Morishima K. KEGG: New perspectives on genomes, pathways, diseases and drugs. *Nucleic Acids Res*. 2017;45(D1):D353-D361. doi:10.1093/nar/gkw1092
[6] Croft D, Mundo AF, Haw R, et al. The Reactome pathway knowledgebase. *Nucleic Acids Res*. 2014;42(D1):D472-7. doi:10.1093/nar/gkt1102
[7] Orchard S, Ammari M, Aranda B, et al. The MIntAct project--IntAct as a common curation platform for 11 molecular interaction databases. *Nucleic Acids Res*. 2014;42(D1):D358-63. doi:10.1093/nar/gkt1115
[8] Salwinski L, Miller CS, Smith AJ, Pettit FK, Bowie JU, Eisenberg D. The Database of Interacting Proteins: 2004 update. *Nucleic Acids Res*. 2004;32(Suppl.1):D449-51. doi:10.1093/nar/gkh086
[9] Keshava Prasad TS, Goel R, Kandasamy K, et al. Human Protein Reference Database - 2009 update. *Nucleic Acids Res*. 2009;37(SUPPL. 1):767-772. doi:10.1093/nar/gkn892
[10] Han H, Shim H, Shin D, et al. TRRUST: A reference database of human transcriptional regulatory interactions. *Sci Rep*. 2015;5(11432):1-11. doi:10.1038/srep11432
[11] Jorba G, Aguirre-Plans J, Junet V, et al. In-silico simulated prototype-patients using TPMS technology to study a potential adverse effect of sacubitril and valsartan. *PLoS One*. 2020;15(2):e0228926. doi:10.1371/journal.pone.0228926
[12] Kirkpatrick S, Gelatt Jr. CD, Vecchi MP. Optimization by Simulated Annealing. *Science (80-)*. 2007;220(4598):671-680. doi:10.1126/science.220.4598.671
[13] Collet P, Rennard J-P. Stochastic Optimization Algorithms. In: *Intelligent Information Technologies*. ; 2008:1121-1137. doi:10.4018/978-1-59904-941-0.ch064
[14] Bishop CM. *Pattern Recognition and Machine Learning (Information Science and Statistics).*; 2006. https://www.springer.com/gp/book/9780387310732.
[15] Russell SJ, Norvig P. *Artificial Intelligence A Modern Approach.*; 2010. doi:10.1017/S0269888900007724
[16] Anellis IH. Peirce's truth-functional analysis and the origin of truth tables. *Hist Philos Log*. 2012;33(1):87-97. doi:10.1080/01445340.2011.621702
[17] Wishart DS, Knox C, Guo AC, et al. DrugBank: A knowledgebase for drugs, drug actions and drug targets. *Nucleic Acids Res*. 2008;36(SUPPL. 1):901-906. doi:10.1093/nar/gkm958
[18] Kuhn M, Letunic I, Jensen LJ, Bork P. The SIDER database of drugs and side effects. *Nucleic Acids Res*. 2016;44(D1):D1075-D1079. doi:10.1093/nar/gkv1075
[19] Jorba G, Aguirre-Plans J, Junet V, et al. TPMS technology to infer biomarkers of macular degeneration prognosis in in silico simulated prototype-patients under the study of heart failure treatment with

sacubitril and valsartan. *BioRxiv*. 2019:625889. https://doi.org/10.1101/625889.

[20] Anaxomics Biotech S.L. Biological Effectors Database. TPMS technology. http://www.anaxomics.com/biological-effectors-database.php. Published 2019. Accessed July 31, 2019.

[21] Herreros-Villanueva M, Perez-Palacios R, Castillo S, et al. Biological Relationships between miRNAs used for Colorectal Cancer Screening. *J Mol Biomark Diagn*. 2018;09(04). doi:10.4172/2155-9929.1000398

[22] Deng J, Park D, Wang M, et al. BCL2-BH4 antagonist BDA-366 suppresses human myeloma growth. *Oncotarget*. 2016;7(19):27753-27763. doi:10.18632/oncotarget.8513

[23] Cartron PF, Gallenne T, Bougras G, et al. The first α helix of Bax plays a necessary role in its ligand-induced activation by the BH3-only proteins bid and PUMA. *Mol Cell*. 2004;16(5):807-818. doi:10.1016/j.molcel.2004.10.028

[24] Raslova H, Vainchenker W, Plo I. Eltrombopag, a potent stimulator of megakaryopoiesis. *Haematologica*. 2016;101(12):1443-1445. doi:10.3324/haematol.2016.153668

[25] Leroy E, Defour JP, Sato T, et al. His499 Regulates Dimerization and Prevents Oncogenic Activation by Asparagine Mutations of the Human Thrombopoietin Receptor. *J Biol Chem*. 2016;291(6):2974-2987. doi:10.1074/jbc.M115.696534
