# Supplementary material for: Elucidating the Mechanism of Action of the Attributed Immunomodulatory Role of Eltrombopag in Primary Immune Thrombocytopenia: An In Silico Approach
Source: Int J Mol Sci. 2021 Jun 27;22(13):6907. doi: 10.3390/ijms22136907 (PMC8269123; doi:10.3390/ijms22136907)
Supplement: Supplementary file 1 [file ijms-22-06907-s001.zip › Supplementary figures.pdf]

SUPPLEMENTARY FIGURES

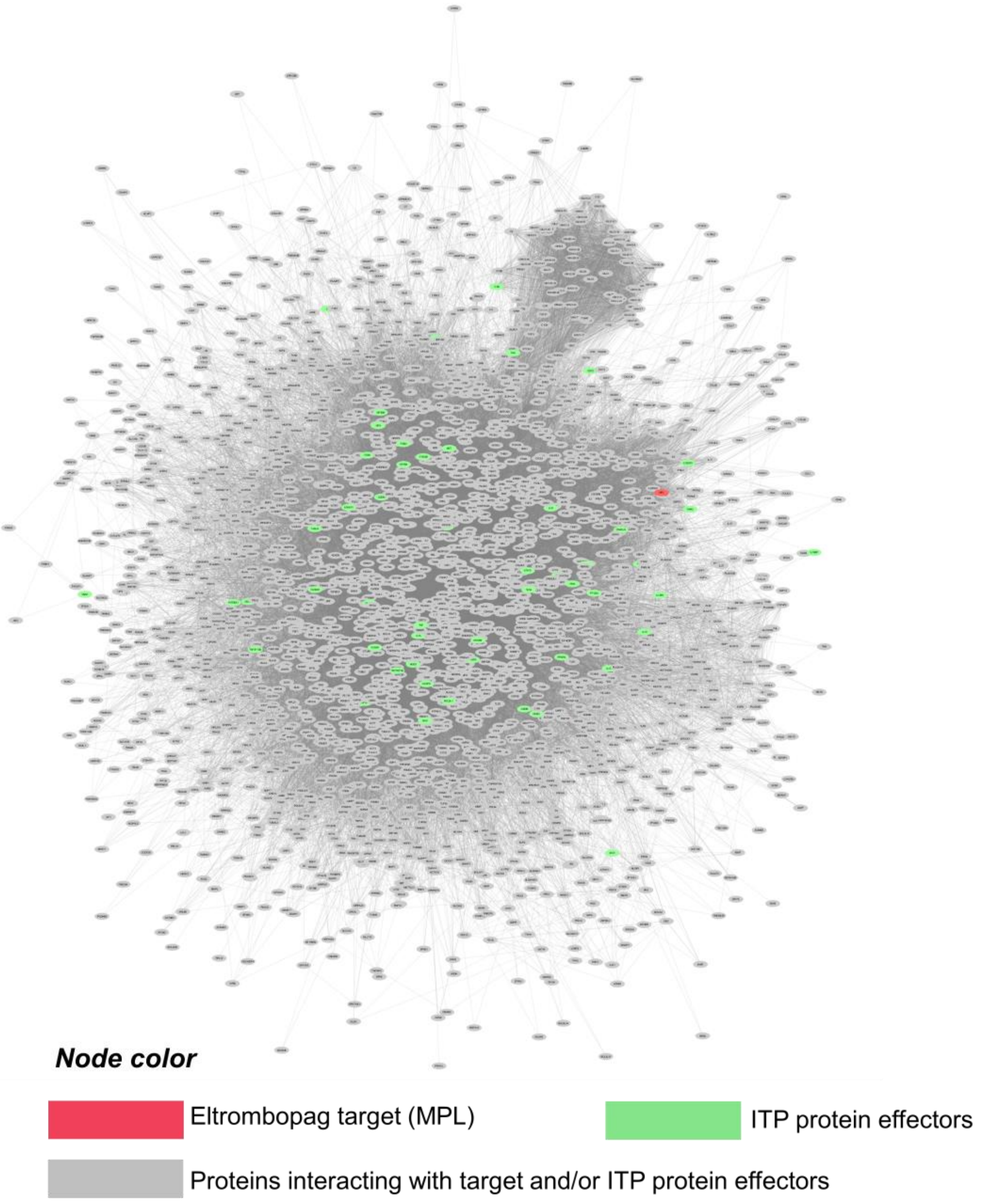

Figure S1. ITP interactome. Protein-Protein interaction network around ITP key proteins.

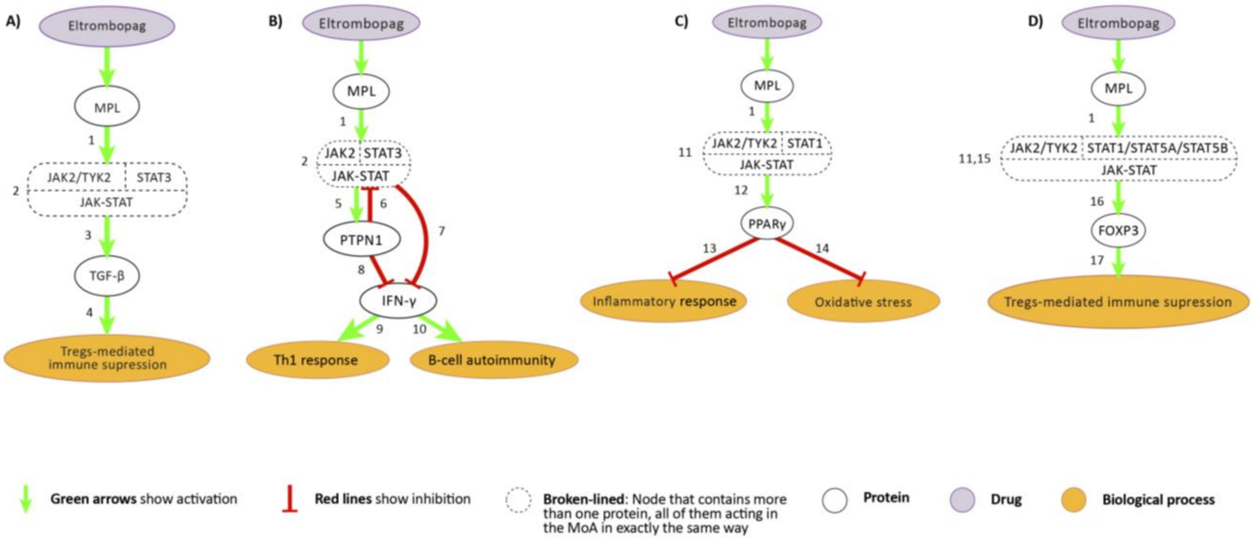

Figure S2. Schematic representation of predicted eltrombopag mechanisms of action over ITP key proteins (linked to their functions in ITP). The number on each link indicates to the number of the corresponding entry on Supplementary Table S5, Supplementary File 1, to retrieve the sources of information found in the scientific literature supporting the predicted mechanisms.
